# Robotic world models—conceptualization, review, and engineering best practices

**DOI:** 10.3389/frobt.2023.1253049

**Published:** 2023-11-02

**Authors:** Ryo Sakagami, Florian S. Lay, Andreas Dömel, Martin J. Schuster, Alin Albu-Schäffer, Freek Stulp

**Affiliations:** Institute of Robotics and Mechatronics, DLR (German Aerospace Center), Wessling, Germany

**Keywords:** world model, state representation, software architecture for robotics and automation, environment modeling, autonomous robots

## Abstract

The term “world model” (WM) has surfaced several times in robotics, for instance, in the context of mobile manipulation, navigation and mapping, and deep reinforcement learning. Despite its frequent use, the term does not appear to have a concise definition that is consistently used across domains and research fields. In this review article, we bootstrap a terminology for WMs, describe important design dimensions found in robotic WMs, and use them to analyze the literature on WMs in robotics, which spans four decades. Throughout, we motivate the need for WMs by using principles from software engineering, including “Design for use,” “Do not repeat yourself,” and “Low coupling, high cohesion.” Concrete design guidelines are proposed for the future development and implementation of WMs. Finally, we highlight similarities and differences between the use of the term “world model” in robotic mobile manipulation and deep reinforcement learning.

## 1 Introduction

The term “world model” (WM) has been used to signify many different concepts. Over 30 years ago, the term was used to denote a software component that internally represents the state of the world (Triggs and Cameron, 1991). Already in the 1970s, Shakey the robot had such a component (Nilsson, 1984). More recently, the deep learning community has adopted the term to describe internal simulators that an agent uses to simulate the development of the world (Taniguchi et al., 2023).

Given the generality of the term “world model,” it has essentially become a homonym for quite distinct concepts. The aim of this review article is to consolidate and refine the terminology for WMs and their subcomponents and classify existing approaches. We believe this is essential to enable researchers to clarify what type of WM they are referring to and facilitate the motivation for the design and implementation of WMs.

The main focus of this article is on WMs for physical robots that execute actions to perform tasks. Within this scope, a concise definition is given by Bruyninckx (2021): *“world model: the information the robot has about the world around it, and that needs to be shared between several activities.”* Here, “about the world around it” implies that the world is constrained to the local physical world in which the robot executes its actions. “The information the robot has about the world” implies that the WM reflects the external physical world, and sensory data are, thus, important to gather this information. The “activities” are internal processes such as planning and decision making. This implies that the internal WM must be a representation rich and accurate enough to select goal-directed actions and perform tasks.

**FIGURE 1:**
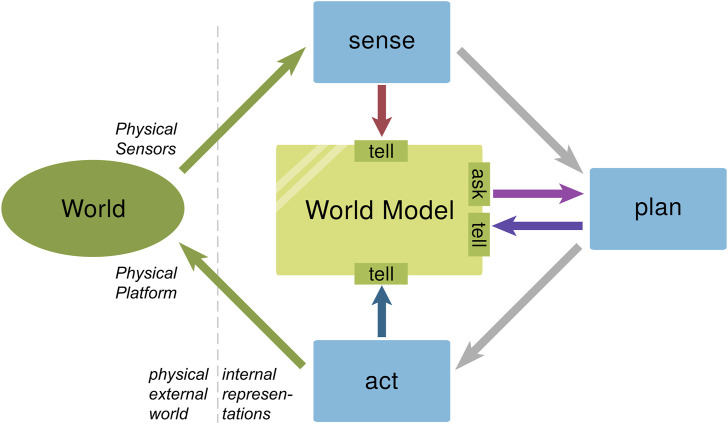
A WM is an internal representation that reflects relevant parts of the physical world in which the robot operates. It is shared by multiple sensors, planners, and actors.

From a software engineering point of view, “shared between several activities” in the aforementioned definition is important. Classically, the robot’s activities are categorized into three types: sense, plan, and act (Gat et al., 1998). This implies that a WM is a software component that integrates information from multiple sensors, planners, and actor components and provides consolidated information about the state of the world to the same components. Information is exchanged with a WM at its interfaces, which are generically categorized into two types: tell and ask (Tenorth and Beetz, 2013). As described in Section 4, information is added or modified through the “tell” interfaces and queried through the “ask” interfaces, as shown in Figure 1.

We argue that storing and sharing information among components is the main motivation for implementing WMs. More concretely, we propose that the software engineering principles of “Design for use,” “Do not repeat yourself,” and “Low coupling, high cohesion” should guide which states and operations should be included in a WM, and even whether one is required in the first place. The aforementioned point also highlights the software engineering perspective on WMs taken in this paper. However, we also describe the more recent deep learning perspective on WMs and show how our design principles apply to and motivate WMs in a deep learning context also.

There are early (Angelopoulou et al., 1992) and more recent (Landsiedel et al., 2017) reviews on robotic WMs. The nomenclature and some of the common concepts of world modeling were presented by Belkin et al. (2012). We consider the main contributions and added value of this review to be 1) to propose a terminology for common subcomponents of a WM; 2) to identify typical properties and design dimensions of WMs, as well as their underlying rationale; 3) to provide an up-to-date overview of concrete WMs in robotics, as well as their classification along the design dimensions identified; and 4) to elucidate principles and guidelines for design decisions about the organization and implementation of these components.

An in-depth motivation and a model-based approach of WMs are provided in the insightful and ongoing “Building Blocks for the Design of Complicated Systems featuring Situational Awareness” (Bruyninckx, 2021). Our review provides a software-engineering perspective and aims to be more concise by focusing on WMs.

The rest of this review is structured as follows. We first bootstrap our terminology using the Kalman filter as a simple and well-known example in Section 2. To provide an intuition of robotic WMs used in real-world environments, and to demonstrate the applicability of our terminology to such complex WMs as well, we introduce two more use cases in Section 3. Based on that, we elaborate on the main components of a world model—its boundary, state representations, and operations—in Sections 4–6, respectively. Using the terminology introduced, we then conduct case studies and classify 15 works on WMs in Section 7. We then describe the underlying principles for the design and implementation of WMs in Section 8 and conclude with Section 9.

## 2 World model language bootstrapping

Before turning to complex robotic WMs, we bootstrap our terminology using a simple, well-known illustrative example: the Kalman filter (Welch and Bishop, 1995). Kalman filters iteratively estimate the mutable state $x\in R^{n}$ by incorporating information of the control inputs $u\in R^{l}$ and measurements $z\in R^{m}$ with the following model^1^:

$$x_{k+1}=Ax_{k}+Bu_{k}+w\;\text{Time}\;\text{update}\;\text{model},$$ (1)

$$z_{k}=Hx_{k}+v\;\text{Measurement}\;\text{update}\;\text{model}.$$ (2)

**FIGURE 2:**
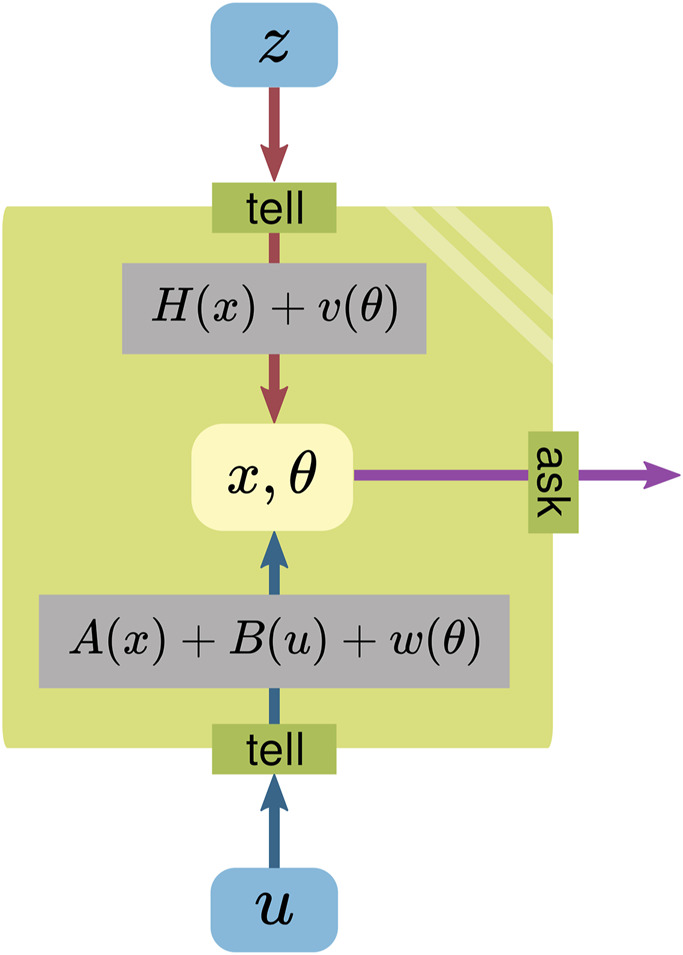
Model of the Kalman filter illustrated as a WM, consisting of a state (which holds a mutable **x** and a constant parameter ***θ***), operations (using **A**, **B**, and **H**), and a boundary.

Figure 2 reinterprets these formulas in terms of the WM template in Figure 1.

As a WM, the *state*, consisting of a *mutable* **x** and a *constant parameter* ***θ***, is meant to reflect the real world. The multiplications with the matrices **A**, **B**, and **H** are *operations* applied to the state to integrate/extract information. The **v** and **w** could be seen to originate from ***θ***, which is used for the operations.

The *WM boundary* is represented by the tell/ask interfaces; everything inside of it is considered to belong to the WM. Information can be provided to the WM via the tell interface, while the ask interface is used to extract information.

Based on the aforementioned analyses, we define the components of a WM as follows.

Definition 1 A WM is segregated by a boundary and internally consists of a state and operations.

## 3 Introductory examples of robotic world models

Having bootstrapped our terminology with the Kalman filter example, we now apply it to more complex robotic WMs. This demonstrates that the terminology is applicable to different robotic WMs. Two systems from different domains are analyzed to provide a first intuition of what a WM is in a real robotic system. Section 7 lists 15 case studies using our complete terminologies that are described in Sections 4–6.

### 3.1 NeBula system

**FIGURE 3:**
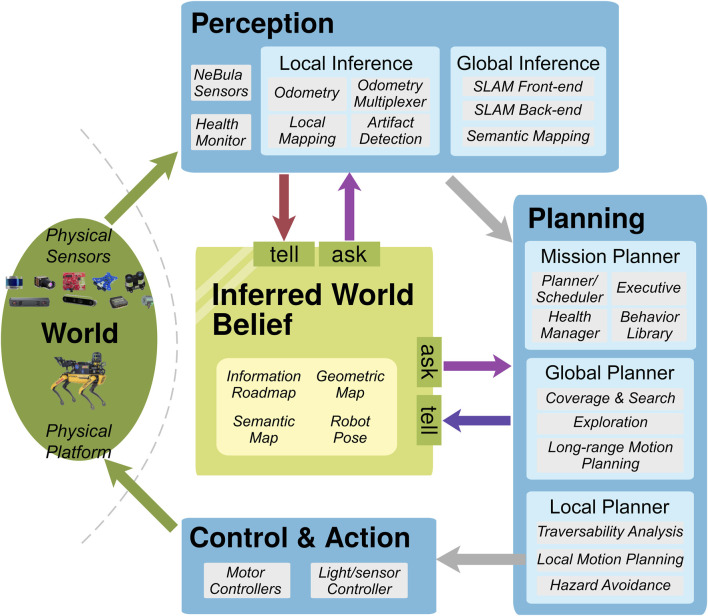
Architecture of the NeBula system [adaptation of Figure 6 from the study by Agha et al. (2021)].

The NeBula system (Agha et al., 2021) is a framework for autonomous robotic exploration of unknown extreme environments. The framework was deployed on a team of multiple different mobile robots, which participated in the DARPA Subterranean Challenge^2^ and completed a task of reaching, detecting, recognizing, and localizing artifacts in various subterranean environments. Figure 3 shows a partial depiction of the architecture; this figure is adapted from Figure 6 from the study by Agha et al. (2021) according to our WM concept shown in Figure 1.

The *inferred world belief* plays the role of the WM in this framework. It integrates the information acquired through the perception pipelines and provides relevant information for planning activities.

The state inside the WM consists of the maps of the environment as well as the robot pose. Different state representations are employed depending on the information to represent; the point clouds and occupancy grids are used for the geometry, while the graph-based representation (called the information roadmap) is used for coverage and traversability.

The simultaneous localization and mapping (SLAM) components convey a major part of the information to the WM. After preprocessing the raw sensor inputs and asking the most recent state from the WM^3^, the perception components first infer the local map as well as the robot pose and then propagate the results into the global scene. The planning components also convey the information to the WM; they estimate the traversability and the hazard of the surrounding area based on the most recent map queried from the WM.

### 3.2 KnowRob system

**FIGURE 4:**
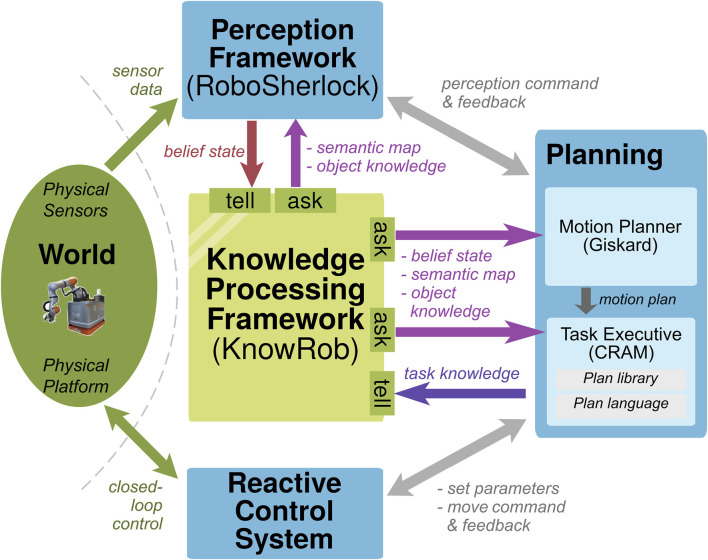
Architecture for the REFILLS project, where KnowRob (Tenorth and Beetz, 2013) is used as the WM [adaptation of Figure 6.1 from the study by Cavallo et al. (2022)].

KnowRob (Tenorth and Beetz, 2013; Beetz et al., 2018) is a knowledge processing system for autonomous robots to perform manipulation tasks. KnowRob not only stores original, high-resolution, continuous data but also provides abstracted knowledge through semantic annotations. The framework has been deployed on different mobile manipulation robots and enabled them to perform pick-and-place tasks in indoor environments. Figure 4 shows a partial depiction of its architecture and has been adapted to our template from Figure 6.1 from the study by Cavallo et al. (2022).

As a central knowledge processing system, KnowRob maintains a *belief state* of the world; i.e., it serves as a WM. It employs the Resource Description Framework as a representation and stores data as subject–predicate–object triples. The ask interfaces are called *computables*, which are used to answer queries. Queries can ask not only to extract information from the state but also to compute new information not stored in the state using an ontology as prior knowledge.

Using sensor data, the perception framework RoboSherlock (Beetz et al., 2015) tells new states to the WM based on the most recent semantic map and the object knowledge. The interface is triggered by a high-level task description as a command.

The task executive CRAM (Winkler et al., 2012) and the motion planner Giskard (Fang et al., 2016) also ask the most recent belief state, the semantic map, and the object knowledge for planning. The executed task knowledge is conveyed to the WM, allowing the system to learn from episodic memories.

## 4 World model boundary

In Section 3, we demonstrated the applicability of Definition 1 to more complex robotic use cases. Sections 4–6 elaborate on the concepts of each constituting part of a WM: a boundary, a state, and operations.

The boundary of a knowledge base is generically defined by two types of interfaces: tell and ask (Tenorth and Beetz, 2013). The tell interface is used for providing new information, e.g., experiences and beliefs based on new sensor data, to a robot’s memory, while the ask interface is used for querying inferred knowledge, e.g., to enable informed decision making during task execution. In this section, we first elaborate on these tell/ask interfaces from the perspective of robotic WMs and argue (in Section 4.3) that the boundary is not inherent to the system but a design decision.

### 4.1 Tell interface

The tell interface is the only way to pass information to the WM. It defines where and which data can be passed to the WM. It does not necessarily provide direct access to the state.

Since components can provide information only through the tell interface, it offers a lot of very different inputs to the WM. Depending on the design, it may take low-level data such as raw images or very high-level data such as Planning Domain Definition Language (PDDL) symbols (Fox and Long, 2003). We identified the following types of information that are typically passed to the WM (examples are given in the parentheses):

• Sensor data (images)
• Interpreted sensor data (object poses)
• Planning and simulation data (PDDL symbols)
• Action data (expected outcomes by task execution)
• Data from other knowledge bases (experience representations)
• Data from outside (human operator knowledge, enterprise management system)

A WM does not necessarily provide all of the aforementioned information; depending on its requirements, only a subset of them is usually provided on the tell interface.

It should be noted that the state usually stores only relevant data abstracted through the tell interface. This implies that not all information provided on the tell interface can be retrieved again on the ask interface.

### 4.2 Ask interface

The ask interface is the only way to obtain information from the WM. It defines which data can be retrieved from the WM while not necessarily providing direct access to the state.

Since components can query information only through the ask interface, it also provides very different outputs from the WM. Information that can be retrieved from the WM includes the following types (examples are given in the parentheses):

• Geometric information (transformations between objects)
• Physical information (load data at an end effector)
• Qualitative information (which objects are on the table?)
• Semantic information (which is “my” cup?)

The ask interface often enables information to be retrieved that is not directly passed via the tell interface, because the WM itself can derive new information from what it is told and has stored in its state by applying operations (see Section 6). This is already the case for the minimal example of the Kalman filter, where **x** can be asked, but only **z** and **u** are told.

### 4.3 Design dimension of the world model boundary

**FIGURE 5:**
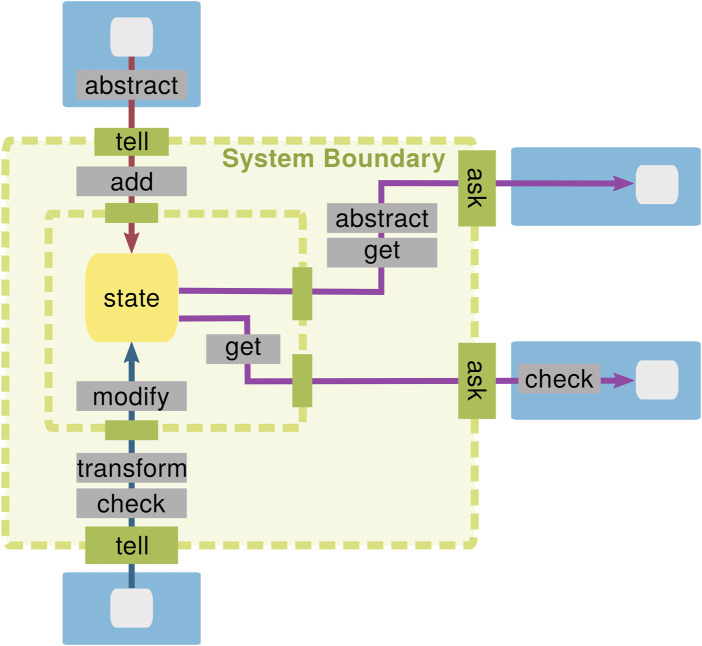
Two examples of different WM boundaries (dashed green lines) that could be defined for a WM.

When components tell/ask information to/from the WM, multiple different operations could be combined and applied on the information along the dataflow (“operations” are explained in Section 6). Therefore, given a robotic system, there are numerous ways to draw a line to segregate the operations within and outside of the WM, as shown in Figure 5. This means that the tell/ask interface can be defined at any locations, and where to set them is a design decision of the robot architecture. Design guidelines for this aspect are discussed in Section 8.2.

## 5 State representation in world models

Within the WM, we make a distinction between the *state* of the world and the *operations* that update this state. This interpretation is best understood in the context of object-oriented programming (OOP), where an object has an internal state represented by its instance variables, which are typically private to avoid direct access. This internal state is what we refer to as the WM state in this article. The tell/ask interface defines the public member functions the WM should implement to provide access to its state. Within the WM, *operations* can modify the internal state (see the gray boxes in Figure 5) but cannot be called directly from outside the WM, which are, thus, represented by private member functions. Properties of the WM state are presented in this section, and those of the WM operations are presented in Section 6.

### 5.1 State representation: robot or environment, concrete or abstract

In mobile robotics, we consider a physical mobile robot operating in a physical environment^4^. Together, they constitute the physical world that the robot has to reflect in its WM.

In many WM representations covered in this review, there is a (partial) separation between the state representation of the *robot* itself and its *environment*. This is possible because the robot and the environment are physically separated. This increases reusability, as separating the environment and robot models means that the latter can be easily reused in different environments.

The state itself is always virtual in a sense that it is an internal representation of the real world. However, there is a separation between the *concrete* part that reflects the physical reality (e.g., a cup on a table) and the *abstract* part that does not (e.g., a grasp pose for this cup). The design goal of reusability again motivates this separation; concrete aspects will be valid for different tasks or scenarios, while some abstract aspects can only be used for specific cases.

**FIGURE 6:**
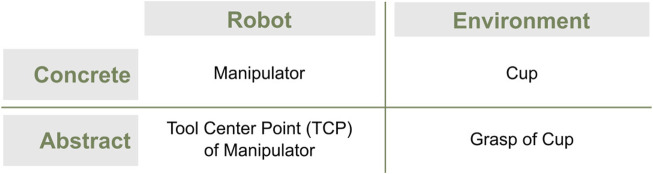
Examples of parts of the state according to the terminologies robot/environment and concrete/abstract.

Figure 6 illustrates examples of different parts of the states according to the robot/environment and concrete/abstract aspects. A robot with a manipulator whose task is to grasp a cup often needs to model the manipulator, its tool center point (TCP), the cup, and its grasp. The manipulator is a concrete property of the robot, whereas the TCP of the manipulator is set abstractly by designers to make task programming easier. The cup is a concrete object in the environment, whereas potential grasps for the cup are specific to the manipulator to perform the task.

Making these distinctions explicit, both conceptually and in the software, makes WMs more modular and reusable for different robots, environments, and tasks.

### 5.2 State representation: permanent or transient, given or estimated

For all practical purposes in robotics, gravity on Earth can be assumed to be permanent. Walls in houses generally stay where they are. Furthermore, for most robots, the length of their rigid body links will not change^5^. Assuming that such aspects of the world will not change, i.e., that they are *permanent*, has many advantages. It means that such aspects can be relied on even in different situations once they are given by designers or estimated by the robots. Once the gravity on Earth is provided or obtained, the robots on Earth can always measure the mass of the grasped object from the force measurement. Once the poses of the walls are obtained, the robots can always localize themselves even if humans change the layout of furniture drastically. On the other hand, *transient* parts of the state are assumed to potentially change and, thus, must be estimated and updated.

It is a natural design decision to represent the permanent and transient aspects as *constant parameters* and a *mutable state* within the WM, respectively. However, the mutable state could also represent variables that are assumed permanent. This is typically the case if those values are calculated during the runtime of the robot. If, for instance, a robot navigating a room creates a map of it at runtime, the poses of the walls should be part of the mutable state; even though they are assumed to be permanent in the real world, their estimated values can be updated with better estimates as the robot progresses. In such cases, the mutable state should have an internal structure to distinguish the permanent and transient aspects so that the robot can exploit the aforementioned advantages.

For almost all robotics applications, data are *given* as prior knowledge to the robot WM during its initialization or during the operation. For instance, a mobile robot might be given an initial map before it starts navigating. Such aspects that do not need to be estimated and, instead, are determined offline can be provided to the robot, e.g., in a static file. On the other hand, it is quite likely, especially for autonomous robots, that they need to *estimate* the (mutable) state of the world by themselves. This is crucial for the robots to operate in dynamic environments where objects move or in partially unknown environments where only a limited amount of information can be given *a priori*.

**FIGURE 7:**
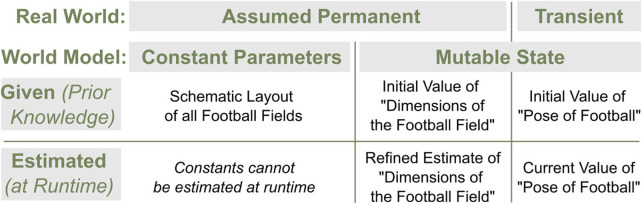
RoboCup (Kitano et al., 1997) examples for parts of the state according to the terminologies permanent/transient in the real world, constant parameters/mutable state in the WM, and given as prior knowledge or estimated at runtime.

Figure 7 illustrates examples of different aspects of the representation categorized according to the concepts and terminologies introduced so far. A robot whose task is to play football in any football field—as in RoboCup (Kitano et al., 1997)—often needs to model the field and the pose of the ball. Since all football fields share a general rectangular layout with two goals on either side, such permanent knowledge is provided by the designers. If the actual dimensions of the field to be played on are known, they can be given and encoded as constant parameters. If the exact dimensions are not known beforehand, an initial value can be provided as part of the mutable state, which is then refined and updated through estimation. The pose of the ball must be part of the mutable state and continually estimated.

### 5.3 State representation: time

As discussed in Section 5.2, the mutable state of the WM is expected to change over time. Therefore, in this section, we highlight *time* as an important dimension of the mutable state^6^. It is crucial to distinguish two separate types of time here: 1) *the real-world time*, which refers to real-world events or changes and 2) *the belief time*, at which the robot holds a certain belief as part of its WM state. A state can model these two time concepts independently. Modeling time is often useful for error analysis, recovery, and learning-based automatic improvements.

**FIGURE 8:**
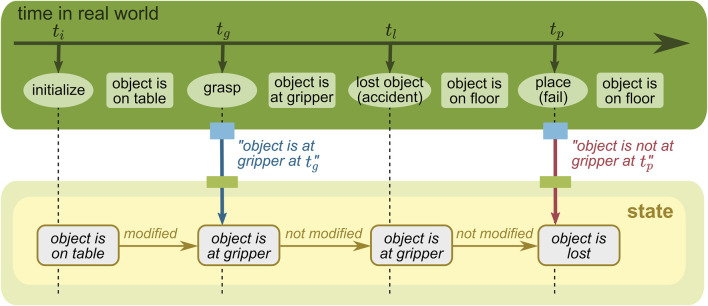
WM state without a time concept.

**FIGURE 9:**
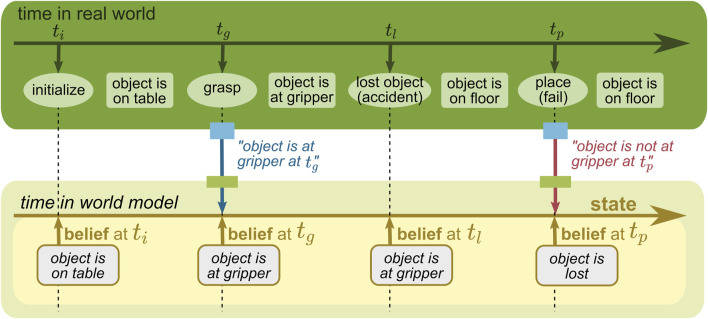
WM state with a time concept of its belief. The beliefs are stored in the state associated with time.

**FIGURE 10:**
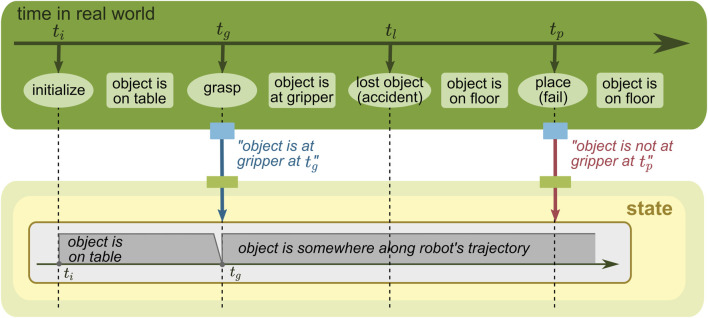
WM state with a time concept of the real world. The belief, modeling changes in the real world with time, changes over time, but only the most recent belief at *t*_*p*_ is stored in the state and shown in the figure.

**FIGURE 11:**
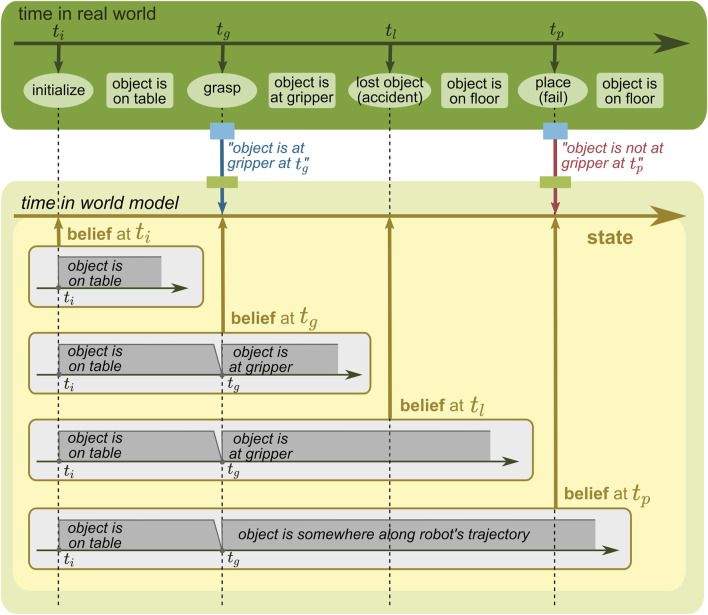
WM state with a time concept of its belief and the real world.

To describe how these two time concepts are different and can be modeled, let us consider a mobile manipulation task for a robot with navigation and manipulation capabilities (see the green rectangle on the top of Figures 8–11). The robot is first initialized next to an object placed on a table (at *t*_*i*_). Then, the robot grasps the object with the gripper (at *t*_*g*_) and navigates to a target location. However, during navigation, the object slips and is dropped on the floor (at *t*_*l*_). The robot does not observe this accident, and as a result, the placing task fails (at *t*_*p*_). The robot has two opportunities to provide information to the WM: when the robot grasps the object and when the robot tries to place the object. The former is provided by an action component that executes the grasping skill, and the latter is provided by a perception component that detects an object in the gripper.

#### 5.3.1 State without time

We first highlight that a WM does not always need to model time. For many use cases, it is sufficient to have a state that only holds the most recent estimate of its belief about each relevant fact of the real world. In the example given in Figure 8, the belief about the object location changes over time when new information is provided, but only the most recent belief is stored and maintained. Although it simplifies the model, it loses the past information, and thus, it could not guess the object location at *t*_*p*_ anymore.

#### 5.3.2 State with belief time

When the state changes, it is possible to model when the change in the belief happens. In the example given in Figure 9, the state changes similarly to Figure 8, but each belief stored is associated with time. This concept enables the state to model *when the robot thought* about the real world. Due to this advantage, the robot in this example could query its past belief, e.g., “when the robot held a belief at *t*_*l*_, where was the object then?” (the answer is “in the gripper”).

#### 5.3.3 State with real-world time

Another time concept is about changes in the real world. In the example given in Figure 10, the state stores the information reflecting the concrete events; the object is estimated to be on the table in [*t*_*i*_, *t*_*g*_ ] and at somewhere along the robot trajectory in $\left[t_{g}\right.$ . This concept enables the state to model *when the real world changed*. By taking this advantage, for example, poses of a constantly moving object can be estimated by inter/extrapolation.

#### 5.3.4 State with both belief and real-world time

Since these two time concepts are independent of each other, they could be modeled together, as shown in Figure 11. In this case, the state keeps its belief at different moments, each of which models the time in the real world. To understand this easier, it should be noted that changes in the state over time are additionally modeled compared to Figure 10; for instance, the most recent belief given in Figure 11 matches the state given in Figure 10. The combination of these two time concepts enables the WM to answer complicated questions, e.g., “when the robot held a belief at *t*_*l*_, where was the object at *t*_*g*_, and is that belief still up-to-date?” (the answer is “the object was believed to be in the gripper but it was updated afterward at *t*_*p*_, and it is now actually believed to be somewhere along the robot trajectory”).

#### 5.3.5 Literature analyses

Time is not represented in the studies by Nilsson (1984), Lomas et al. (2011), Leidner et al. (2014), Milliez et al. (2014), Dömel et al. (2017), and Lehner et al. (2018). Some WMs such as those studied by Triggs and Cameron (1991), Blumenthal et al. (2013), Foote (2013), Tenorth and Beetz (2013), Beetz et al. (2018), Schuster et al. (2018), Schuster (2019), and Agha et al. (2021) represent time in the state. However, the conceptual distinction of the belief time and the real-world time is not always considered explicitly. To the best of our knowledge, no WM in the literature employs both the belief and the real-world time together. The concepts introduced in this section would help WM designers be more conscious of and consistent with which time aspects they implement in the state.

## 6 World model operations

While the previous section described the design dimensions of the WM state, this section does so for the operations that manipulate the state. The input arguments of an operation are information from the tell interface and/or the state itself and/or the output of other operations; examples were previously given in Figure 5 in gray. The output of an operation is returned through the ask interface, included in the state, or passed as an input to another operation (see also Figure 5).

General classes of operations defined in this section are used in Section 7 to analyze different WM implementations and in Section 8.2.2 to discuss the rigorous design of WMs.

### 6.1 Basic operations

Based on the aforementioned generic definition, a set of basic operations can be defined. All operations can be categorized to either one of them or a combination of them.

#### 6.1.1 Add

First, we introduce a set of operations that can write into the state^7^. The *add* operation adds input data to the state. Therefore, after executing this operation, the input is part of the new state, meaning that this operation implements a direct write-access to the state. It should be noted that this is the minimal requirement to the *add* operation. Depending on the design of the WM, the *add* operation might be more complex. For instance, sanity-check operations might be integrated to guarantee that the new state conforms to certain rules (e.g., there must be no redundancy in the state).

#### 6.1.2 Remove

The *remove* operation removes input data from the state. After execution of this operation, the data are deleted from the state and not contained in there anymore. Thus, as the *add* operation does, the *remove* operation also has a direct write-access to the state. It should be noted that the *remove* operation might further modify other parts of the state than the input depending on the design. For example, if the state is represented as a tree and the *remove* operation is applied to a node, it might remove all children of the node as well.

#### 6.1.3 Modify

The *modify* operation replaces previously stored information with new information. This is technically equivalent to the combination of the *add* and *remove* operations. Semantically, however, the modification of a world state is more than a combination of the *add* and *remove* operations. Since the state reflects the real world, a modification of the state implies that either the world has changed or the belief about the world has been incorrect.

Depending on the design, the *modify* operation might be the only operation which is allowed to write to the world state. For instance, a controller usually has a vector of a constant dimension as a state. All measurements (inputs) modify the state by replacing outdated measurements and do not allow the removal/addition of an element from/to the vector.

#### 6.1.4 Get

From here, we introduce a set of operations that can read from the state^8^. The *get* operation returns a subset of the state as an output. Therefore, this operation implements a direct read-access to the state without modifying it.

#### 6.1.5 Abstract

The *abstract* operation reduces information from an input and/or the state to an output by abstracting the information. Since information is lost through this operation, it is not possible to reconstruct the original information from the result.

For example, we consider the geometric relation between two objects; it is possible to abstract a qualitative relation, e.g., on, as an output, using their pose transformation as an input and a threshold of the distance as a parameter. However, even with the information that one object is on another, it is not possible to reconstruct the pose transformation between them.

#### 6.1.6 Transform

The *transform* operation transforms an input and/or the state to an output. The difference to the *abstract* operation is that no information is lost, and thus, there exists an inverse operation. For example, we consider a situation where a robot localizes an object pose with a movable camera, and we want to obtain the object pose with respect to a robot base. To calculate it, a *transform* operation can be used based on the object pose relative to the camera as an input and the pose of the camera relative to the robot base from the state. The *transform* operation is not limited to geometric transformations; a unit conversion (e.g., to transform data from millimeter to meter) is another example.

### 6.2 Compound operations

If a WM chooses its boundary close to its state, the aforementioned basic operations are sufficient to implement the tell/ask interface. For many WMs, however, compound operations are necessary to integrate the information from the tell interface into the WM state. Likewise, compound operations enable the ask interface to retrieve information that requires interpretation based on the WM state.

These compound operations can be composed of basic operations. The basic operations can be chained to allow a more complex interpretation of the data (*pipeline concept*). With this concept, the output of a basic operation is used as an input of the next operation. Instead of piping the data through operations, basic operations can also be used to make decisions (*decider concept*). In this case, the output of the basic operation is used not to modify the incoming data but to determine how to integrate them or how to extract further information. In the following, we show two examples of such compound operations, one on the tell interface and the other on the ask interface.

**FIGURE 12:**
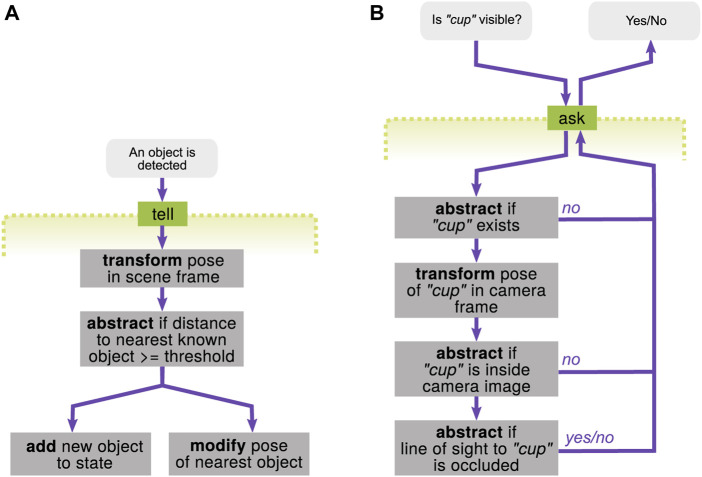
Examples of compound operations. **(A)** Integrating an object detection result into the state via the tell interface. **(B)** Querying if a unique, specific cup is visible to the robot through the ask interface.

Tell interface—object detection with a camera: The first example is to integrate new information of the object detection (see Figure 12A). When a perception component estimates a pose of an object with a camera, the pose is first estimated in the camera coordinate frame and given to the tell interface. This information could be interpreted either 1) as a detection of a new object instance or 2) as a refinement of the pose of a known object instance. This decision can be made, e.g., by calculating if the distance to the nearest known object exceeds a certain threshold. Therefore, the basic *transform* and *abstract* operations are piped through, and then, either the *add* or *modify* operations are decided to be triggered.

Ask interface—checking if the cup is visible: Another example is to query if a unique, specific cup is visible to the robot or not (see Figure 12B). The computation to answer this query could be a sequence of steps with increasing computational effort. First of all, it checks if the cup already exists in the state. If yes, the pose of the cup needs to be checked if it is within the field of view of the camera (using the *transform* operation beforehand). If this is also yes, the last step is to check if the line of sight to the cup is occluded by other objects or not. If either one of the aforementioned checks (using the *abstract* operations) returns “no,” the following (computationally expensive) operations can be skipped and could answer “no” at the ask interface.

## 7 Case studies

The two use cases in Section 3 aimed at providing concrete examples to illustrate what a robotic WM is. This section now interprets 15 WM implementations—including the two aforementioned—by applying the terminology introduced so far. Our aims are to: 1) show that our concepts are general enough to be applicable to WMs from very different domains; 2) motivate the need for a common terminology as the terminologies used now are different in each work; 3) provide an overview of four decades of work on robotic world models; and 4) classify these works (see Table 1).

**TABLE 1:** Classification of representative robotic world models.

World models	System coupling	Interface types	Robot in a state	Permanent aspects in a state	Model time	Complex operations	Perspective	Robot type
Shakey the robot (Nilsson, 1984)	Low	Few	Yes	Yes	No	No	System	Mobile robot
Oxford World Model (Triggs and Cameron, 1991)	Low	Few	No	No	No	No	Framework	Mobile manipulator
ROS TF (Foote, 2013)	High	Few	No	Yes	Yes	No	Framework	General robot
AIMM system (Dömel et al., 2017)	High	Many	Yes	No	No	Yes	System	Mobile manipulator
LRU1 (Schuster et al., 2020, Schuster et al., 2018, Schuster, 2019)	High	Few	Yes	Yes	Yes	Yes	System	Mobile robot
LRU2 (Lehner et al., 2018)	High	Many	Yes	Yes	Yes	Yes	System	Mobile manipulator
NeBula system (Agha et al., 2021)	High	Few	Yes	Yes	Yes	Yes	System	Mobile robot
KnowRob (Tenorth and Beetz, 2013, Beetz et al., 2018)	High	Many	Yes	Yes	Yes	Yes	Framework	Service robot
Rollin’ Justin (Leidner et al., 2014; Leidner et al., 2012, Bauer et al., 2018)	High	Many	Yes	Yes	No	No	System	Mobile manipulator
Robot Scene Graph (Blumenthal et al., 2013)	Low	Few	No	Yes	Yes	Yes	Framework	Mobile manipulator
Multi-robot teams (Roth et al., 2003)	Low	Few	Yes	No	Yes	No	System	Mobile robot
SPARK Systems (Lehner et al., 2018, Warnier et al., 2012)	Low	Few	Yes	No	No	Yes	Framework	Service robot
DL: World Models (Ha and Schmidhuber, 2018)	Low	Few	Yes	No	No	No	Framework	Agent in computer games
DL: DayDreamer (Wu et al., 2022)	Low	Few	Yes	No	No	No	Framework	Mobile or manipulation robot

System coupling means how many components communicate with the WM. Interface types are considered to be many if a wide range of different, heterogeneous data are provided/received at the tell/ask interfaces. Perspective types categorize the referenced work by a perspective they describe the WM from, i.e., as a component within a system or as a framework.

### 7.1 Shakey the robot

Already in the first mobile, autonomous robot, Shakey (Nilsson, 1984), the WM was a central component. Based on this WM, the robot could plan its actions independently and represent the state of itself and its environment.

#### 7.1.1 WM boundary

The *WM boundaries* are defined by four functions. On the *tell interface*, there are three functions, ASSERT, DELETE, and REPLACE. The *ask interface* is implemented by the function FETCH. The interfaces of the Shakey WM have variable distance to the WM *state*. Some information can be added directly to the *state*, e.g., a robot position, but for others, *compound operations* are necessary, e.g., locations of movable objects.

#### 7.1.2 State representation

In Shakey, the *state* is represented by formulas of first-order logic. The *state*, thus, consists of statements which link entities from five different entity classes (Faces, Doors, Rooms, Objects, and Robot) to other entities or values via various predicates. The applicable predicates depend on the entity class. The *state* models the *environment* as well as the *robot* information, and it models *concrete* as well as *abstract* information (e.g., problematic locations). The *state* consists of *parameters* and *mutable states* that are either *given* or *estimated* by the system. The *state* does not represent *time*.

#### 7.1.3 Operations

The *tell/ask interface* is similar to the *basic operations add*, *remove*, *modify,* and *get*. For some information, these can also be used directly to implement the *interface*. However, in addition to the *state*, there are axioms that define the *operations* on the *state*. For example, adding a location for an object can either *add* a new statement to the *state*, or if a location is already stored, *modify* the existing one, since the rule exists that an object can only be at one location. For some parts of the *state*, the *interface* is, therefore, realized by *compound operations*.

### 7.2 Oxford World Model

The Oxford World Model (Triggs and Cameron, 1991) is proposed as a geometric database of an autonomous navigation robot for a factory environment.

#### 7.2.1 WM boundary

One of the main motivations of the Oxford World Model is to encourage a clean and modular internal software architecture where different components communicate via the WM. Therefore, the *WM boundary* is set so that the WM provides a uniform inter-module *interface*.

#### 7.2.2 State representation

The following types of information are stored in the *state*: a) a factory layout and route maps; b) object positions and their 3D models; c) sensor features; d) caches of local or temporary data; and e) tasks and scheduling information. It represents the *environment* aspect for both the *concrete* part (such as poses and 3D models) and the *abstract* part (such as sensor features). The factory layout and route maps are *permanent* but stored into the *state* together with other information. Each type of these data is attached with a common header containing a timestamp. Although this time is described to model a “time-of-last-modification” of objects, it is not explicit if this models *belief time* or *real-world time*.

#### 7.2.3 Operations

*Basic operations* such as *adding* and *removing* objects are executed via indices. In addition, generalized *compound operations* are provided, i.e., to perform a specific *modification* on every object having the same object type, or to *get* objects satisfying a specific spatial condition.

### 7.3 ROS TF

The Robot Operating System Transform Library (ROS TF) (Foote, 2013) provides a standard way of maintaining coordinate frames and transforming data on the open-sourced middleware ROS (Quigley et al., 2009). The main motivation is to free the programmers from the necessity of calculating the transformation between frames.

#### 7.3.1 WM boundary

Due to its general, low-level scope as a use case, the *WM boundary* is set around the *state*. *Operations* needed for *transform* computations are included within the *WM boundary*, and many components require such a calculation interface with the ROS TF. More complex *operations* (such as how to utilize obtained transforms) are expected to be implemented on external components *telling/asking* the WM.

#### 7.3.2 State representation

The ROS TF uses a tree structure; nodes represent a frame (a coordinate system), and edges represent a 6-degrees of freedom (DOF) relative pose. Since the main purpose is to calculate the transformations between frames, they do not distinguish the *robot/environment, concrete/abstract* aspects explicitly.

For efficiency, it distinguishes the *mutable state* and *constant parameters* by using different representations, /tf and /tf_static, respectively. While the /tf maintains transformations with the *real-world timestamps* and represent changes in the real world, the /tf_static expects that any transform is static and considered to be valid at any time.

#### 7.3.3 Operations

The *basic get* and *add operations*, as well as the *transform operation*, are implemented. For sending (*adding*) information, the sendTransform *operation* is used. For querying (*getting*) a transformation between two frames, the lookupTransform *operation* is used, in which the *transform operations* are chained. Using the spherical linear interpolation (SLERP) algorithm (Shoemake, 1985) (for orientations) and a linear inter/extrapolation (for positions), lookupTransform approximates the changes of frames given a timestamp.

### 7.4 AIMM system

DLR’s Autonomous Industrial Mobile Manipulator (AIMM) (Dömel et al., 2017) focuses on performing transportation tasks autonomously in partially unstructured factory environments with its mobile platform and manipulator.

#### 7.4.1 WM boundary

The AIMM employs a navigation system, an object detector, a motion planner, and a flow-control system. They must share the world state, and thus, the *WM boundary* can be drawn between them. The object detector provides information about the pose, category, size, and detection quality of objects at the *tell interface*. The motion planner, on the other hand, queries information about rigid body kinematics such as free space and collision at the *ask interface*. The flow-control system exchanges information with the WM at both the *tell and ask interfaces*. The *ask interface* is typically used for making decisions depending on the current *state*. Once the flow-control system executes a skill, the *tell interface* is used to reflect the high-level effects expected by the execution.

#### 7.4.2 State representation

The representation focuses on providing not a detailed geometric description but a topological-level description of the relation between different types of objects. For this purpose, the WM *state* employs a tree representation. Each node represents an object with labels specifying its types, and edges connecting nodes represent a 6-DOF transformation^9^. Nodes representing the *robot* have a type Robot, which are distinguished from the *environment* aspects. The object ownership information (such as which object is grasped by the manipulator), which is *abstract*, is modeled by edges constructing the parent–child relations. Other *abstract* aspects, e.g., grasps and approaches for manipulation, are modeled as child nodes of a *concrete* node with a type RigidBody. Since components require only the most recent *state*, *time* is not modeled.

#### 7.4.3 Operations

There are *basic operations* having the write access to the *state*, such as to *add* a new node, *remove* an existing node (and its children), *modify* the property of a node or the 6-DOF pose of an edge, and to reassign a node to another node.

To have the read access to the *state*, there are not only *basic operations* to simply *get* a node (or its properties) but also *compound operations*. One of them is to query a pair of nodes satisfying certain conditions by utilizing the tree structure. By specifying types and/or properties of nodes as well as a maximum/minimum number of hops between them, only a relevant subset of the *state* is effectively extracted. For instance, the motion planner queries only Robot and RigidBody objects under the branch of the current scene node.

### 7.5 LRU1

The core WM on DLR’s Lightweight Rover Unit 1 (LRU1) robot (Schuster et al., 2020) is the multi-layer and multi-robot SLAM system (Schuster et al., 2018; Schuster, 2019).

#### 7.5.1 WM boundary

The *WM boundary* can be drawn on the input side between typically stateless low-level perception components, such as stereo matching, and the modules of the SLAM system itself that contain the *state* related to local and global localization and mapping, such as the keyframes of visual odometry, the state vector of a local reference filter, the pose graph for global optimization, the local obstacle maps for navigation, and the global 3D maps for exploration planning and multi-agent coordination. Thereby, the SLAM WM receives preprocessed sensor data from proprioceptive sensors such as inertial measurement units (IMUs) and exteroceptive sensors such as stereo cameras via its *tell interface*. On the output side, the *WM boundary* can be drawn between the SLAM system providing pose and map information via its *ask interface* and the typically higher-level components consuming these, such as path planning, exploration planning, mission coordination, or visualization modules.

#### 7.5.2 State representation

Sensor properties, such as noise models, sensor calibrations, and certain environment characteristics, such as the local gravity constant, are assumed to be *permanent* in the real world during the system runtime and, thus, provided as *parameters* to the WM. The *state* variables contain information about the *concrete* aspects of the world, such as robot poses and 3D geometry, as well as *abstract* annotations, such as submap poses, which are mainly used for SLAM-internal purposes. *Real-world time* is used in the local estimation both for sensor data acquisition timestamps as well as state prediction timestamps, which are important to relate measurements and estimates to events in the environment and to the measurements and estimates by other robots. As the SLAM is concerned with moving agents, non-static poses are always accompanied by *real-world timestamps*. For the resulting maps, timestamps are, however, not necessarily required as the consuming modules are only interested in the most up-to-date model of the environment available.

#### 7.5.3 Operations

Most complex *operations* integrating measurements or estimates into the *state* variables are triggered by the *tell interface*. In contrast, most *operations* triggered by the *ask interface* are simple *get operations* to return parts of the *state*, accompanied by minor *abstractions* and *transformations*. However, expensive complex computations, such as global map compositions, could also be triggered on demand to *abstract* or/and *transform state* variables and *modify* caches for intermediate results. Perception data are typically *pushed* into the WM, and the WM itself not only *pushes* out new *state* estimates (e.g., poses and maps) to consumers but also provides *pull*-based interfaces, e.g., in inter-robot communication to provide missing data to other agents demanding them.

### 7.6 LRU2

DLR’s Lightweight Rover Unit 2 (LRU2) robot (Schuster et al., 2017; Schuster et al., 2020), being a mobile manipulator, uses two separate WMs, one for navigation, similar to the LRU1 (see Section 7.5), and an additional one to support mobile manipulation tasks, similar to the AIMM (see Section 7.4). The information required for manipulation tasks are substantially different from that used by navigation and exploration components. Therefore, following the “Low coupling, high cohesion” principle (see Section 8.1.3) as well as the guideline given in Section 8.2.1.2, a WM focusing on quasi-static scene representation is additionally employed. Regardless of the difference of the application domain (from the factory automation to the planetary exploration), the AIMM WM can be employed with ease due to its generally designed *boundary*.

There is a unique data exchange on the *WM boundary* between these two WMs. The navigation WM queries the real-world dynamics of manipulated objects at the *ask interface* of the AIMM WM. For example, when LRU2 manipulates a box with fiducial markers, the navigation WM requires to be informed of the changes accordingly since objects with the markers are used as landmarks for the SLAM system.

### 7.7 NeBula system

As described in Section 3, NeBula is an autonomy solution for navigation, mapping, and exploration of unknown extreme environments (Agha et al., 2021).

#### 7.7.1 WM boundary

The purpose of the system is similar to the LRU1 (see Section 7.5), and thus, the WM also has many similarities, which can also be implied by the “Design for use” principle (see Section 8.1.1). Similar to the LRU1, the *WM boundary* can be drawn at the output of the low-level, stateless sensor-fusion component HeRO and at the input of high-level planning components.

#### 7.7.2 State representation

The *state* models belief over the internal *robot state* such as its pose, as well as the external surrounding *environment state* such as maps and hazards. The *state* is an abstracted representation of not only spatial information but also *temporal* information. This allows for maintaining probability distributions over various *state* domains. As a unique representation of this system, the *state* includes the hierarchical belief representation called the Information Roadmap (IRM) for making decisions on where to explore, taking uncertainty into account. The IRM is a graph representation containing *concrete* information such as occupancy and *abstract* information such as coverage and traversal risks.

#### 7.7.3 Operations

Similar to the LRU1, complex *operations* are mostly for integrating inputs from the odometry source and perception results (such as loop closures) at the *tell interface*. As one of the *compound operations*, factor graph optimization employs a method to identify and discard incorrect loop closures that can arise while working in environments with poor perceptual conditions. At the *ask interface*, the *operations* are mostly basic ones to *get* part of the information from the *state*.

### 7.8 KnowRob system

As described in Section 3, KnowRob is a knowledge processing system specifically designed for autonomous robots to perform manipulation tasks in various environments (Tenorth and Beetz, 2013; Beetz et al., 2018). Its primary function is to maintain a belief state of the world, serving as a WM that helps the robot understand and interact with its surroundings. It features a modular architecture that interfaces components such as the perception framework RoboSherlock (Beetz et al., 2015; Bálint-Benczédi et al., 2019), the task executive CRAM (Beetz et al., 2010), and the motion planner Giskard (Fang et al., 2016).

#### 7.8.1 WM boundary

The *WM boundary* of KnowRob is not tightly confined around the belief *state*. Instead, it encompasses various kinds of *state representations* and *operations* that interact with the belief *state*. Information can be asserted through *tell interfaces* to the belief *state* from different components such as RoboSherlock (Beetz et al., 2015; Bálint-Benczédi et al., 2019) or CRAM (Beetz et al., 2010). The *ask interfaces* are also queried by RoboSherlock (Beetz et al., 2015; Bálint-Benczédi et al., 2019), CRAM (Beetz et al., 2010), and Giskard (Fang et al., 2016).

#### 7.8.2 State representation

KnowRob predominantly employs a sophisticated symbolic and semantic representation of the belief *state*, which encompasses both the *robot* and its *environment* in a cohesive manner. The semantic concepts of ontology can be regarded as *parameters*, while the instantiated data within the belief *state* can be considered *mutable*. Moreover, *time* plays a significant role, particularly in the context of episodic memory knowledge. However, no explicit distinction is made between the *real-world occurrence timestamps* and the *robot’s belief update timestamps*. Additionally, the belief *state* does not discriminate between *concrete* and *abstract* information explicitly, but both representations are collectively represented in the knowledge graph. Nonetheless, the system features a component that primarily focuses on symbolic *abstract* data, as well as an *inner WM* that represents the *concrete state* through a physics simulation.

#### 7.8.3 Operations

On both the *tell* and *ask interfaces*, *operations* are used to query the belief *state* and assert information to it. The *operations* that query the knowledge base range from *basic operations* to *compound* ones, depending on the reasoner (ProLog, SPARQL, … ) used. Additionally, further *compound operations* are implemented as computables that can query the belief *state*.

### 7.9 Rollin’ Justin

The WM of the humanoid robot Rollin’ Justin serves as a fundamental element in the hybrid planning and reasoning methodology, as detailed by Leidner et al. (2014).

#### 7.9.1 WM boundary

The *WM boundary* for both the *tell* and *ask interfaces* can be tightly drawn close to the world *state*. For example, the localization component updating the robot’s position within the world *state* based on a pre-computed environmental map communicates through the *tell interface*. Another perception component leverages fiducial markers to adjust the geometric pose of detected objects via the *tell interface* during execution. The hybrid planner functions as both a planning and acting module, utilizing the *tell interface* to modify the symbolic state representation during execution while also querying via the *ask interface* the current symbolic world state for task planning purposes. The motion planner similarly queries via the *ask interface* the geometric world state, thus enabling the hybrid planning approach.

#### 7.9.2 State representation

The *state representation* adopts an object-centric perspective of the environment, associating both geometric and symbolic information with each object. Within the hybrid planning approach implemented on Rollin’ Justin, PDDL predicates are necessary for high-level task planning, while the geometric state representation facilitates geometric planning. The object database (Leidner et al., 2012; Leidner, 2017) serves as a knowledge repository containing information on object affordances and appearances, embodying a combination of *assumed permanent parameters* in the form of an object taxonomy and *transient, mutable state*, such as object-specific *abstract* grasp frames. The world representation component (Leidner, 2017) comprises the *mutable state* variables and is instantiated from this knowledge.

#### 7.9.3 Operations

*State* information access relies primarily on *basic operations*. The aforementioned components directly *add*, *remove*, or *modify* both geometric and symbolic state representations. Within the world representation component, geometric and symbolic representations are modeled independently. However, various external approaches exist to ensure consistency between these representations (Bauer et al., 2018; Bauer et al., 2020; Lay et al., 2022).

### 7.10 Robot Scene Graph world model

Blumenthal et al. (2013) proposed a Robot Scene Graph (RSG) WM as a central, complete geometrical 3D representation of autonomous robots.

#### 7.10.1 WM boundary

The goal of this WM is to centralize knowledge from the planning, perception, control, and coordination components. The *WM boundary* is set quite away from the *state*. As described in the following sections, the WM takes raw sensor data at the *tell interface* and caches intermediate data. On the *ask interface*, for instance, components can query collision models of the entire scene.

#### 7.10.2 State representation

The *state* uses a directed acyclic graph (DAG) to represent a full 3D scene. In addition to a unique ID and a list of attributes (which are *abstract*), each node holds a bounding collision geometry to represent the *concrete, environment* aspects. Any geometric data are *assumed to be permanent* and stored as *parameters*. Poses are represented not by the edges but by nodes called transform. Since these transforms tend to *vary* over time, each transform node stores its data with associated timestamps to represent the *real-world time*. The *time* concept is utilized for *operations* for tracking and prediction.

The *state* stores not only the final resulting data but also raw sensor data and intermediate data created by *operations*.

#### 7.10.3 Operations

The unique IDs are used for providing *basic operations* such as setData to *add* information and getData to *get* information. As an example of *compound operations*, the RSG WM provides an *operation* to calculate spatial information. Given a starting node and a targeted node, the *operation* queries a path connecting these two nodes. Based on the transform nodes along the path, it connects *transform operations* in a *pipeline* manner. Since each node can have multiple parent nodes, it is possible that multiple paths are discovered. In this case, different policies could be employed, e.g., either to use the path containing the most recent timestamp or to use semantic tags to decide a most reliable path.

### 7.11 World model for multi-robot teams

Roth et al. (2003) proposed a robotic WM for a team of robots under high communication latency. Since the application domain is to let the team of robots play soccer in RoboCup (Kitano et al., 1997), the WM is quite specific to this domain. Nevertheless, it still addresses unique challenges common to any dynamic environments for a multi-robot setup.

#### 7.11.1 WM boundary

The WM takes two sources of information at the *tell interface*: vision and communication. The vision component detects all relevant objects in the world by their colors and produces information about pose in the local coordinate frame of the robot. The communication is possible but with only limited bandwidth and high latency. Therefore, the robots neither synchronize data streams nor have a centralized external server, and instead, they communicate only relevant data on an as-needed basis. At the *ask interface*, planning components query information for decision-making activities.

#### 7.11.2 State representation

Each robot maintains its individual world *state* and a global world *state*. The individual one contains the *robot, concrete* aspects such as the robot’s position and heading, as well as the *environment, concrete* aspects such as positions of teammates and opponents. As *parameters*, an *a priori* map of the field is given initially and utilized for localizing the robot. The global world *state* consists of the *environment, concrete* aspects such as the location of the teammates and the ball, as well as the *environment, abstract* aspects such as if a robot is the goal keeper and if a robot saw the ball.

#### 7.11.3 Operations

As in the study by Stulp et al. (2010), the individual *state* is updated by three different policies: either from vision information, from communicated information, or combination of both. The updates to the location of the robot and the opponents come directly from the vision information. The location of the teammates is given by the communicated information. The ball position is estimated by using both sets of information via a Kalman filter.

### 7.12 SPARK system

SPAtial Reasoning and Knowledge (SPARK) (Milliez et al., 2014) is a WM that maintains a state of the world to enable a robot to plan and act, especially collaborating with human workers. The goal is to generate symbolic facts from the geometric *state representation*, allowing for the creation of collaborative plans and generation of efficient dialog.

#### 7.12.1 WM boundary

The WM gathers geometric information from perception components at the *tell interface* and provides symbolic information about the robot’s belief about human beliefs at the *ask interface*. The symbolic information is utilized by the human-aware symbolic task planner and the execution controller (Warnier et al., 2012).

#### 7.12.2 State representation

In the *state*, it maintains the *robot* pose, the quasi-static objects in the *environment*, and the positions of humans including their hands, head, and shoulders. The *state* is mostly concerned with the *concrete* aspects, and only minor *abstract* features are represented for checking the identity of humans and objects. No *time* is modeled, and the *state* assumes that the most recent belief holds true if no further information is available.

#### 7.12.3 Operations

There are several complex *operations* employed in SPARK. The first example is to track and manage object locations in the environment. Using the *decider concept*, it combines *basic operations* and either *modifies* the pose of the object, *removes* the object from the *state*, or keeps the *state* without any change. An important *compound operation* is to estimate human beliefs, e.g., to compute the visibility and reachability of an object from a human perspective.

### 7.13 Deep learning: introducing world models

The term “world model” was first introduced to the deep learning community by Ha and Schmidhuber (2018), where the WM refers to a compressed spatial and temporal representation of the environment. The work focuses on learning latent spaces as abstractions of the input sensor space so that it can be used as an internal simulator, which builds on very early work on learning such models (Schmidhuber, 1990).

#### 7.13.1 WM boundary

In the architecture proposed by Ha and Schmidhuber (2018), the world model boundary can be drawn around the vision (V) and the memory (M) networks. The V network serves as a gateway for sensory input, consuming RGB images, which can be considered the *tell interface*. The M network takes as input the embeddings from the V network as well as the actions from the control (C) network. Since the world model boundary in their paper is drawn around the V and M networks, this propagation of actions from the C network back to the M network can also be considered a *tell interface*. The embeddings from both the V and the M network serve as input to the C network, and one can interpret this as the *ask interface*.

#### 7.13.2 State representation

The *state* consists of the network embeddings of both the V and M networks and is *mutable*.

#### 7.13.3 Operations

Both the V and M networks can be viewed as *operations*. The V network can be interpreted as an operation that *abstracts* the input 3D image into a latent state representation. Another *abstraction* happens in the recurrent structure of the M network.

### 7.14 Deep learning: DayDreamer

Wu et al. (2022) introduced a WM that learns a forward model of the environment, predicting the environment dynamics. The neural network is trained based on a replay buffer of past sensory inputs to the architecture.

#### 7.14.1 WM boundary

The boundary can be drawn tightly around the neural network where sensory information such as RGB, depth, proprioceptive joint readings, and force sensor information are passed as input to the WM. This sensory input is passed to an encoder network which can be viewed as a *tell interface*. The actor–critic algorithm learns a policy using the latent representation of the encoder network provided through a *ask interface*. There is another *ask interface* in the form of a decoder network that decodes the same latent representation to human-readable information.

#### 7.14.2 State representation

The state representation is represented as the latent encoding of sensory information and predictions from previous states based on the learned forward model. Therefore, the *state* is *mutable*.

#### 7.14.3 Operations

The different components in the neural network architecture can be interpreted as *operations*. The encoder network *abstracts* the sensory input and *adds* information to the latent state representation. The dynamic network acts as a complex operation that applies kind of a projection, i.e., the forward model to the latent state representation, and the reward network uses the latent state representation to predict future rewards. The encoder network operates as an *abstraction*, taking the sensory input and *adding* to the latent state representation. Acting as another operation, the dynamic network performs an additional *abstraction* in the form of a projection to future dynamics of the environment. Finally, the reward network adds an additional layer of *abstraction* to the latent state representation to forecast future rewards.

### 7.15 Deep learning: vision

Another recent interpretation of the “world model” was given by LeCun (Author anonymous, 2022; LeCun, 2022) in a “proposal for a modular, configurable architecture for autonomous intelligence.” It should be noted that this work presents a vision for modularization of deep learning modules for autonomous machine intelligence, which has not been implemented. We include it nevertheless, as it highlights a trend toward the formalization of WMs, as shown in Figure 1.

The WM is one of the six main modules of a configurable architecture for autonomous intelligence. The WM “is a kind of simulator of the part of the world relevant to the task at hand.” LeCun, thus, emphasized the use of WMs as internal simulators more than using them to reflect the actual state of the outside world. Indeed, the state itself is not stored in the world model module but in a module called “short-term memory.” In our terminology, the “short-term memory” would rather be included in the WM boundary.

## 8 Discussion: world model design

As highlighted by the case studies, the design space for WMs is huge. This raises many practical questions for their design and implementation. Which types of queries will the planning components need in order to generate their plans? Which aspects of the world are to be considered transient or permanent, and which of the latter do we know in advance? Should derived intermediate states be cached any time new information is provided or only when a query is made that requires the update? What should be the internal organization of the state? A list, a tree, a relational database, or an ontology?

These questions are difficult to answer generally. They vary based on the robot’s tasks, sensors, and other system components. In this section, we offer guidelines for these design considerations.

### 8.1 Principles from software engineering

First, as a basis for the design guidelines, we highlight the following software engineering design principles that we consider relevant to WM design.

#### 8.1.1 Principle: design for use

Since a WM provides information to other software components, its state should be rich enough to produce the information relevant to these components. This does not mean, however, that the WM should store as much information as possible; there is no reason to store data in the state that will never be used during any tell/ask requests. In the extreme case, if there are no components to ask any information from a WM, there is no reason to have a WM at all, for instance, in the subsumption architecture (Brooks, 1986).

The design of the WM is determined, thus, not by what the designer *assumes* is relevant but what the system *actually* requires. This is the “Design for use” (DfU) principle, which is also used to guide the design of ontologies (Smith et al., 2004). This principle is applicable not only to the contents of the state but also to the operations and the boundary as well.

#### 8.1.2 Principle: do not repeat yourself

As shown in Section 6, deriving the state of the world from multiple sources involves many operations, such as *abstractions* from low-level sensor data to higher-level representations, or *transformations* to convert the data into another form. If different software components implement these operations separately, repetition and redundancy are inevitable. This hinders the reuse of implemented functionality in another component, and furthermore, it makes debugging activity tedious due to the redundancy (Blumenthal et al., 2013). This is applicable not only to the operations but also to the state elements. For example, in the NeBula system (Agha et al., 2021), both the local and long-range motion planning components need the current robot pose, and it would be highly redundant if each consumer implemented its own SLAM algorithm to infer this current pose.

A dedicated WM component should bundle such state elements and operations in one place and enable other components to update and query it so that they must not re-implement duplicated data or redundant computations (Blumenthal et al., 2013). This is the “Do not repeat yourself” (DRY) principle in software engineering, which is formulated as “Every piece of knowledge must have a single, unambiguous, authoritative representation within a system” (Hunt and Thomas, 1999).

#### 8.1.3 Principle: low coupling, high cohesion

The DRY principle helps decide whether a shared WM is required for a robot or not. The “low coupling, high cohesion” (LCHC) principle helps decide how many. This principle states that components in a component-based system should have few couplings between different components and high cohesion within a component (Yourdon and Constantine, 1979) as a tight coupling of components would cause invasive changes on them when modifications are needed (Heim et al., 2015).

As an example of applying the principle, our LRU2 robot (Lehner et al., 2018; Schuster et al., 2020) (see also Section 7.6) is a mobile manipulation platform that navigates based on SLAM and path planning components and manipulates objects using visual pose estimation and sampling-based motion planning. As manipulation is only performed when the robot is not moving, the tasks of navigation and manipulation and, thus, the decision-making components for executing these tasks, are entirely decoupled. It is thus reasonable to have two distinct WMs because the components for navigation and manipulation require disjunct queries on the WM and use different hardware (mobile base vs. articulated arm) and software (the path planner in the 2.5D geometric space for the robot base vs. the motion planner in the configuration space for the manipulator). As the WMs for navigation and manipulation are disjunct, merging them into one WM would lead to a component with low cohesions; therefore, a separation into two is preferable according to the LCHC principle.

If the task specification is changed to require the robot to be able to grasp objects *whilst* moving, this would require a reevaluation of having two WMs. Since the two WMs have to be synchronized quite frequently for such a use case, having two will lead to high coupling of the WMs and, thus, will be considered to be a non-optimal design decision. It is suggested that the two WMs should be merged into one.

### 8.2 Guidelines for world model implementations

The principles introduced in Section 8.1 are applicable to all aspects of the WM: the state representation, the operations, and the boundary. However, these principles sometimes contradict each other, and a trade-off between them must be found. Therefore, the aim of this section is to clarify where such conflicts exist and to concretize more specific guidelines for the implementation of WMs. We propose the following guidelines based on our experiences obtained during the development of the AIMM WM (Dömel et al., 2017), which is described in detail in Section 7.4. Although the guidelines are considered applicable to the WM approaches reviewed in Section 7, there might be robotic systems where the guidelines are not always applicable or not feasible to follow in practice. Nevertheless, the guidelines are our suggestions for supporting design decisions and not strict regulations a WM must always satisfy.

#### 8.2.1 Implementation: state representation

##### 8.2.1.1 Reducing redundancy

If information in a state can be generated by applying operations on other parts of the state, such information is redundant. Since redundancy is a violation of the DRY principle, it should not be cached into the state in principle. The redundancy often makes maintenance difficult; if a part of the state is modified, the corresponding, redundant part must be updated accordingly to avoid information mismatch. However, this guideline often contradicts the “minimize calling frequency and computational load” guideline (Section 8.2.2.2). Thus, the trade-off is between reducing redundancy and computational load.

In the AIMM WM (Dömel et al., 2017), intermediate information is not cached into the state to avoid redundancy since the operations are called mostly in an event-driven manner.

##### 8.2.1.2 Keeping abstraction levels homogeneous

If a single WM must contain information at different levels of abstraction, multiple abstraction levels within the state must be simultaneously kept up-to-date. Having multiple representations within a state, however, makes it difficult to keep the state consistent if the different levels of abstraction represent the same aspect of the world. Ideally, abstraction operations are, thus, computationally inexpensive so that they can be called regularly on demand. This is a specific case of the “reduce redundancy” guideline (Section 8.2.1.1).

In the AIMM WM (Dömel et al., 2017), edges of its tree representation are used only for geometry and not for symbols. If necessary, symbols can be computed via operations without high computational load.

##### 8.2.1.3 Choosing the abstraction level as high as possible, as low as necessary

The appropriate level of abstraction for the state representation is determined by the DfU principle. If the abstraction level is too low, high-level action information cannot be integrated at the tell interface. On the other hand, if the abstraction level is too high, e.g., using only symbolic predicates, a motion planner could not query metric information about the geometry of the world at the ask interface. Thus, an appropriate level of abstraction must be chosen and employed; concretely, it should be as high as possible but as low as necessary.

In the AIMM WM (Dömel et al., 2017), the abstraction level to describe a topological relation between different types of objects is employed so that the sensing components can tell information about detected objects, the planning components can ask collision-free spaces, and the acting components can tell expected effects of skill execution.

##### 8.2.1.4 Decreasing dependency among state elements

Even if a state has a homogeneous level of abstraction without redundancy, a modification in one part of the state may require updates to other parts. For example, we assume a state has a list of object poses with respect to the world coordinate frame. If an object A is placed on another object B in the real world, and if the pose of the object B changes, the state has to update the pose of both objects A and B. Such dependencies among state elements should be avoided as much as possible since they increase the number of operation calls (see Section 8.2.2.2). In this example, employing a tree as a data structure of the state could be one of the solutions [as the AIMM WM does (Dömel et al., 2017)].

##### 8.2.1.5 Using simple time representations

Finding appropriate representations for time in a WM is challenging. As described in Section 5.3, there are two kinds of time. Furthermore, the time in the real world is by nature continuous, while the state and its modification are usually associated with discrete time. Due to such complexity, careless introduction of the time representation could easily increase the model complexity without providing practical advantages. Following the DfU principle, the time representation should be employed only according to the actual necessity from the application. For many WMs, no explicit modeling of time is needed. Instead, they often assume that the current world state represents the current real world.

If modeling of time is required, special attention must be paid to which type of time is represented and how the discretization of time is handled in the model. It is also important to decide carefully which aspects of time to represent in the state and which to implement by operations. For example, the time interval during which an object is at a certain position can be modeled directly in the state. Alternatively, for example, we can store into the state only the moments at which the position of the object has changed and let an operation determine the position at certain points of time by intra/extrapolation. This guideline suggests using a simple representation, and which is simpler depends, thereby, strongly on the application.

In the AIMM WM (Dömel et al., 2017), no time needs to be modeled since having access to the most recent belief about the most recent world satisfies the requirements from components interfacing with the WM.

#### 8.2.2 Implementation: operations

##### 8.2.2.1 Modularizing and reusing

As discussed in Section 6, operations can be combined into compound operations. If some operational routines are common to multiple operations, they should be modularized and reused, following the DRY principle.

In the AIMM WM (Dömel et al., 2017), basic operations are provided as a Python library, allowing for implementing compound operations reusing the basic ones as a module.

##### 8.2.2.2 Minimizing calling frequency and computational load

Calling operations causes computational load. Thus, keeping the call frequency low, especially for computationally expensive calls, is important to achieve better performance.

**FIGURE 13:**
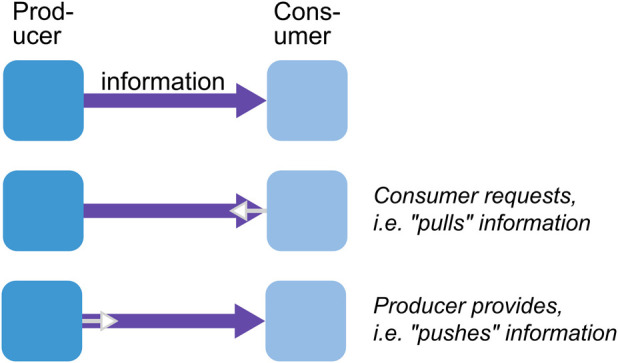
Pushing or pulling information, adapted from Figure 2 from the study by Bainomugisha et al. (2013).

As shown in Figure 13, when operations are triggered, information flows can be decided by either the information source (*producers*) or their dependents (*consumers*) (Bainomugisha et al., 2013)^10^. Producers can *push* information any time it is available, or consumers can request (*pull*) the information when they need it (Bainomugisha et al., 2013). Which way is preferable depends on the use case, i.e., the design decision should be made by the DfU principle. In general, we see that the flow of task-specific information that is only relevant to one or a few consumers is best implemented via a *pull* and the flow of general information that is relevant to many consumers via a *push*.

It should be noted that the *push* and *pull* are implementation models of how operations are triggered and how the tell/ask interfaces are called. Although it is counter-intuitive, for example, the ask interface can be implemented by the *push* approach, where the WM periodically publishes information to other components. Similarly, the tell interface can be implemented by the *pull* approach, where the WM triggers a perception component in a polling manner to update the state periodically.

**FIGURE 14:**
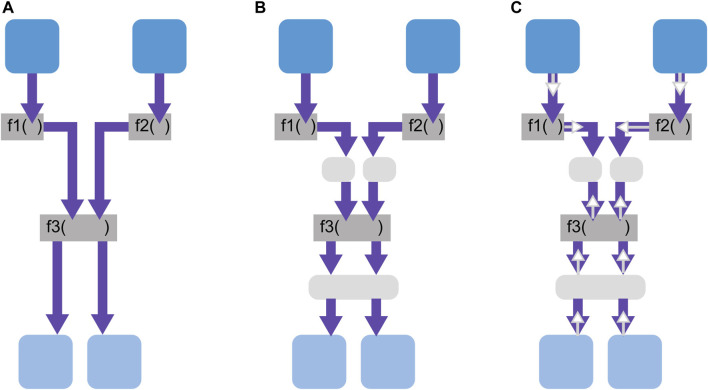
**(A)** Information flow without caching intermediate data in the state. **(B)** State caching results of operations. **(C)** Caching allows for decoupling of push and pull.

As mentioned in Section 8.2.1.1, caching intermediate results in the state causes data duplication. However, it can contribute to minimizing the computation load by operations, as shown in Figure 14A. Without caching, the operations *f*1, *f*2, and *f*3 must be called every time information flows from the producer (at the top) to the consumer (at the bottom). If the results of these functions are cached in the state (gray boxes), they must only be called in case information is out-of-date.

Caching also enables push and pull information flows to be decoupled, as shown in Figure 14C. This means that operations can be implemented to either push or pull information, depending on their frequency and computational cost. We consider, for instance, that *f*1 and *f*2 are computationally cheap and not called very often. Then, any time new information is provided by the producer, it is opportune to immediately push by calling *f*1 and *f*2 and then to store the intermediate result in the state. If *f*3, on the other hand, is computationally expensive, the state would be pushed no further. Rather, *f*3 would only be applied to the cached, pushed results of *f*1 and *f*2 upon request by one of the consumers, and the “pulled” result of *f*3 would be cached as well.

In the AIMM WM (Dömel et al., 2017), as written in Section 8.2.1.1, no intermediate information is cached into the state. Since the WM represents semi-static environments, components basically push information at the tell interface and pull information at the ask interface.

#### 8.2.3 Implementation: boundary

##### 8.2.3.1 Keeping the boundary abstracted from components

If the WM boundary is set too close to the producer and consumer components, operations only specific to them will be included within the WM. Since such component-specific operations are not useful for other components, this is against the DfU principle. This is especially critical when the same WM is used for different robotic systems because of differences in software/hardware components.

The AIMM WM (Dömel et al., 2017) keeps the generality of operations exposed at the boundary, and thus, the same WM can also be used for our planetary exploration rover LRU2 (Lehner et al., 2018) (see also Section 7.6).

##### 8.2.3.2 Setting the boundary where operations are reusable

The aforementioned guideline could lead to an extreme design to have the WM boundary right around the state. However, this leads to reuse of operations; i.e., it is against the DRY principle and Section 8.2.2.1. Thus, the WM boundary should be set so that the operations common and useful for multiple components are included within the WM.

## 9 Conclusion

In this review, we provided an overview of four decades of work on robotic WMs and classified concrete implementations in Sections 3, 7. The main contributions are to make design dimensions of WMs explicit (Section 5, 6) and to highlight the underlying principles that guide decisions on these WM design dimensions (Section 8.1) as well as the trade-offs that may be necessary when they are in conflict (Section 8.2).

We learned that the WM does not exist. Even when following the same guiding principles, good WM designs highly depend on the robotic system, the environment in which it operates, and the tasks it is expected to perform. The principles may well lead to a design where one robot has more than one WM, for instance, one for navigation and another for manipulation, as highlighted in Section 8.1.3.

A current trend is that the deep learning community is increasingly embracing the design of modular neural architectures and has also identified the WM as a necessary component within such designs. This trend—which we believe to be driven by the “low coupling, high cohesion” principle—is very much aligned with the stance in this review, even if the underlying implementation of states and operations as deep neural networks is quite different from the robotic WMs focused on in this review.

In Section 4, we saw that different WM boundaries lead to quite distinct interpretations of what a WM is and what it should encompass. However, all of these interpretations can well be referred to as a “world model,” and it is, thus, essentially a homonym for different concepts. By refining the terminology for WMs and their subcomponents and classifying existing approaches, our aim is to enable researchers to clarify what type of WM they are referring to and facilitate the motivation for their design and implementation.

One of the future research challenges we foresee is how to design a WM for future robotic systems to perform tasks in more realistic, less controlled scenarios. System complexity will increase by employing more novel and diverse hardware/software components, and new requirements will be posed to WMs. We expect the principles and guidelines discussed in Section 8 to promote the invention of novel, unforeseen WM designs.

## Footnotes

1 We dropped the subscripts of **A**, **H**, **w**, and **v** for simplification since the main purpose is to bootstrap the terminology.

2
https://subtchallenge.com/

3 In practice, high-level components of the perception activity contain reasoning activities, which fall into the category of the “planning” in the classical concept, as shown in Figure 1. Therefore, in Figure 3, there is a dataflow from the WM to the perception components via the ask interface.

4 The distinction between a robot and an environment becomes less clear in smart homes or when using Internet-of-things technology, as part of the robot’s sensing capabilities are delegated to the environment. Nevertheless, in mobile robotics, there typically is a robot that can be carried out of the environments. The thing being carried out is what we call the physical robot. The rest is the physical environment.

5 Note that such properties are *assumed* to be permanent but hardly ever *are* intrinsically so. For instance, we do send robots to other planets and moons, renovations in houses do take place, and the kinematics of your robot may change after a hardware upgrade. It depends on the domain which assumptions one is willing to make about aspects that are permanent in the real world.

6 Since the constant parameters never change during the robot operation, the design dimension *time* is by nature not applicable to the parameters.

7 Note that operations can only write into the mutable state since the constant parameters cannot be changed as discussed in Section 5.2.

8 Note that operations can read from both the mutable state and the constant parameters.

9 Although this is similar to the ROS TF (see Section 7.3), the important difference to the ROS TF is that this WM aims to represent the world in a quasi-static, object-based manner with a higher level of abstraction.

10 Note that the WM can be both a producer and a consumer. From the perspective of the tell interface, the WM is a consumer. From the perspective of the ask interface, the WM is a producer.
